# Characterization of myocardial injury phenotype by thermal liquid biopsy

**DOI:** 10.3389/fcvm.2024.1342255

**Published:** 2024-04-04

**Authors:** Karita C. F. Lidani, Robert Buscaglia, Patrick J. Trainor, Shubham Tomar, Alagammai Kaliappan, Andrew P. DeFilippis, Nichola C. Garbett

**Affiliations:** ^1^ Division of Cardiovascular Medicine, Department of Medicine, Vanderbilt University Medical Center, Nashville, TN, United States; ^2^ Department of Mathematics and Statistics, Northern Arizona University, Flagstaff, AZ, United States; ^3^ Department of Chemistry and Biochemistry, New Mexico State University, Las Cruces, NM, United States; ^4^ Molecular Biology and Interdisciplinary Life Sciences Program, New Mexico State University, Las Cruces, NM, United States; ^5^ UofL Health–Brown Cancer Center and Division of Medical Oncology and Hematology, Department of Medicine, University of Louisville, Louisville, KY, United States

**Keywords:** thermal liquid biopsy, myocardial injury, myocardial infarction, unsupervised clustering analysis, longitudinal study

## Abstract

**Background and aims:**

With the advent and implementation of high-sensitivity cardiac troponin assays, differentiation of patients with distinct types of myocardial injuries, including acute thrombotic myocardial infarction (TMI), acute non-thrombotic myocardial injury (nTMi), and chronic coronary atherosclerotic disease (cCAD), is of pressing clinical importance. Thermal liquid biopsy (TLB) emerges as a valuable diagnostic tool, relying on identifying thermally induced conformational changes of biomolecules in blood plasma. While TLB has proven useful in detecting and monitoring several cancers and autoimmune diseases, its application in cardiovascular diseases remains unexplored. In this proof-of-concept study, we sought to determine and characterize TLB profiles in patients with TMI, nTMi, and cCAD at multiple acute-phase time points (T 0 h, T 2 h, T 4 h, T 24 h, T 48 h) as well as a follow-up time point (Tfu) when the patient was in a stable state.

**Methods:**

TLB profiles were collected for 115 patients (60 with TMI, 35 with nTMi, and 20 with cCAD) who underwent coronary angiography at the event presentation and had subsequent follow-up. Medical history, physical, electrocardiographic, histological, biochemical, and angiographic data were gathered through medical records, standardized patient interviews, and core laboratory measurements.

**Results:**

Distinctive signatures were noted in the median TLB profiles across the three patient types. TLB profiles for TMI and nTMi patients exhibited gradual changes from T0 to Tfu, with significant differences during the acute and quiescent phases. During the quiescent phase, all three patient types demonstrated similar TLB signatures. An unsupervised clustering analysis revealed a unique TLB signature for the patients with TMI. TLB metrics generated from specific features of TLB profiles were tested for differences between patient groups. The first moment temperature (*T_FM_*) metric distinguished all three groups at time of presentation (T0). In addition, 13 other TLB-derived metrics were shown to have distinct distributions between patients with TMI and those with cCAD.

**Conclusion:**

Our findings demonstrated the use of TLB as a sensitive and data-rich technique to be explored in cardiovascular diseases, thus providing valuable insight into acute myocardial injury events.

## 1 Introduction

Each year, over 12 million patients present with suspected acute myocardial infarction (MI) to the emergency departments in North America and Europe (1). A systematic review by the Agency for Healthcare Research and Quality of the US Department of Health and Human Services (AHRQ Report) (2) showed that ∼5.7% of emergency department patients receive an incorrect diagnosis, with MI ranking second among conditions associated with the most serious harm due to misdiagnosis.

The etiology of acute MI is complex. Although coronary thrombus overlying a disrupted atherosclerotic plaque is the hallmark and therapeutic target of acute MI, multiple non-thrombotic etiologies, such as coronary vasospasm and demand ischemia, are now known to exist and necessitate different treatments (3, 4). Multiple studies have reported that non-thrombotic MI is at least as common as thrombotic MI (5). While current guidelines distinguish between thrombotic (Type 1) MI and non-thrombotic causes of myocardial injury (4), clinically actionable criteria to distinguish between these two types of myocardial injuries do not exist. Because both types of MI are associated with myocyte injury, they both lead to an increase in circulating levels of troponin, the current gold standard for MI diagnosis. The limitations of current diagnostic strategies are highlighted by the fact that 70% of the ∼6 million US patients presenting to hospital with chest pain are given a benign diagnosis at a cost of approximately $10 billion/year (6–8). Despite the expense of this diagnostic work-up, 2%–5% of patients discharged home with a benign diagnosis are subsequently found to have an acute MI with a worse prognosis than those correctly diagnosed on the initial encounter (6–8). In patients with thrombotic MI, lack of accurate and rapid diagnosis could delay necessary, time-sensitive, anti-thrombotic, anti-coagulant, fibrinolytic, and procedural revascularization therapies, whereas in patients with non-thrombotic myocardial injury, these therapies could lead to unnecessary bleeding/procedural risks without the possibility of clinical benefit (9–11).

**Figure 1:**
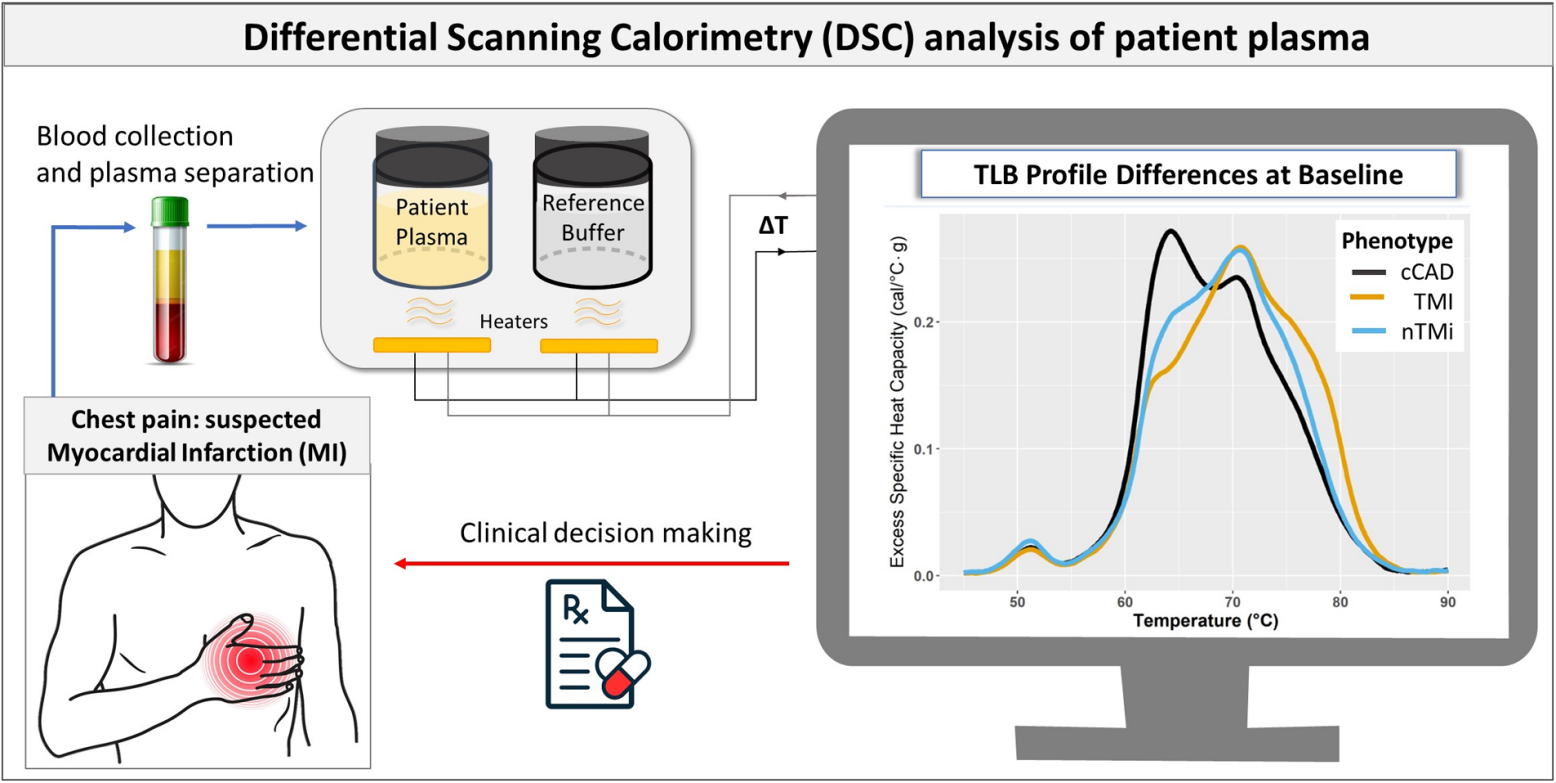
Flowchart illustrating the use of TLB in myocardial injury assessment. The process begins with blood collection and plasma separation in patients with suspected MI. TLB involves DSC analysis to capture the comprehensive protein denaturation behavior of the patient plasma sample. The resulting TLB profiles are used to differentiate between the different forms of myocardial injury (cCAD, TMI, and nTMi), which complements other clinical data in the clinical decision-making process.

Thermal liquid biopsy (TLB) utilizing differential scanning calorimetry (DSC) is a powerful tool that may be applied to characterize and differentiate myocardial injury events, without the need for costly or more invasive procedures. DSC is a thermoanalytical method employed to analyze the heat profiles associated with the denaturation of biomolecules and their interactions with different metabolites. TLB is based on the analysis of non-solid biological tissues (e.g., blood plasma) that captures complex mixtures of heat release and heat absorption that reflect the overall biomolecular makeup of blood plasma at the time of collection (Figure 1). This detects alterations in protein concentration, post-translational modifications, or interaction with other analytes that affect the thermal stability of the plasma proteome (12–14). Previous studies have successfully employed TLB to better understand complex factors contributing to diseases status including cancer (14–21), autoimmune (22–25), and other diseases (15, 26–29). Although TLB offers a comprehensive measure of disease status, with potential for novel characterization and monitoring of diseases, its application in cardiovascular diseases remains unexplored.

Given that atherothrombosis results from an imbalance between thrombotic and fibrinolytic proteins, individual biomolecule measurements may not reflect the complex interplay of multiple biological factors contributing to a pathological state. We hypothesized that TLB may capture the collective biomolecular constitution of blood plasma at the time of sampling, providing a signature TLB profile of acute changes associated with patients with distinct types of myocardial injuries. We sought to characterize TLB at the time of the acute clinical event and quiescent follow-up time points in three patient types: acute thrombotic myocardial infarction (TMI), acute non-thrombotic myocardial injury (nTMi), and chronic coronary atherosclerotic disease (cCAD) (the stable underlying disease necessary for acute TMI). This approach represents a novel use of TLB in the assessment of acute myocardial injury events.

## 2 Materials and methods

### 2.1 Study design and patient recruitment

This investigation is a prospective cohort study to evaluate the utility of TLB for differentiating myocardial injury subtypes. Patients with suspected acute myocardial injury (TMI and nTMi) and suspected cCAD were recruited from two hospitals in Louisville, KY, USA, between September 2014 and January 2020. The study was approved by the University of Louisville Internal Review Board (IRB #14.0437) and both participating hospitals. All patients provided written informed consent.

Patient interviews and medical records were used in the collection of pertinent medical history, physical, electrocardiographic, histological, biochemical, and angiographic data. Coronary angiograms were assessed in a blinded fashion with standardized criteria by the Johns Hopkins Quantitative Angiographic Core Laboratory (30). Laboratory data (troponin I, creatinine, blood cell, and platelet counts) were obtained from the treating hospital clinical laboratory and research blood samples were collected and processed at standardized study time points: baseline/time of invasive angiogram (T0) and 2 (T2), 4 (T4), 24 (T24), and 48 (T48) h post angiogram (unless the patient was discharged from the hospital prior to this time point). In addition, troponin I levels were measured using Beckman Access assay from T0 to T48 to assess peak troponin relative to the upper reference limit (URL of 0.04 ng/ml). Follow-up history, physical exam results, laboratory data and research blood samples were collected at a single follow-up (Tfu) visit 3–12 (median, 3.98) months after the procedure or hospitalization for acute myocardial injury, when the patient was in a stable condition.

### 2.2 Analytical cohort

**Figure 2:**
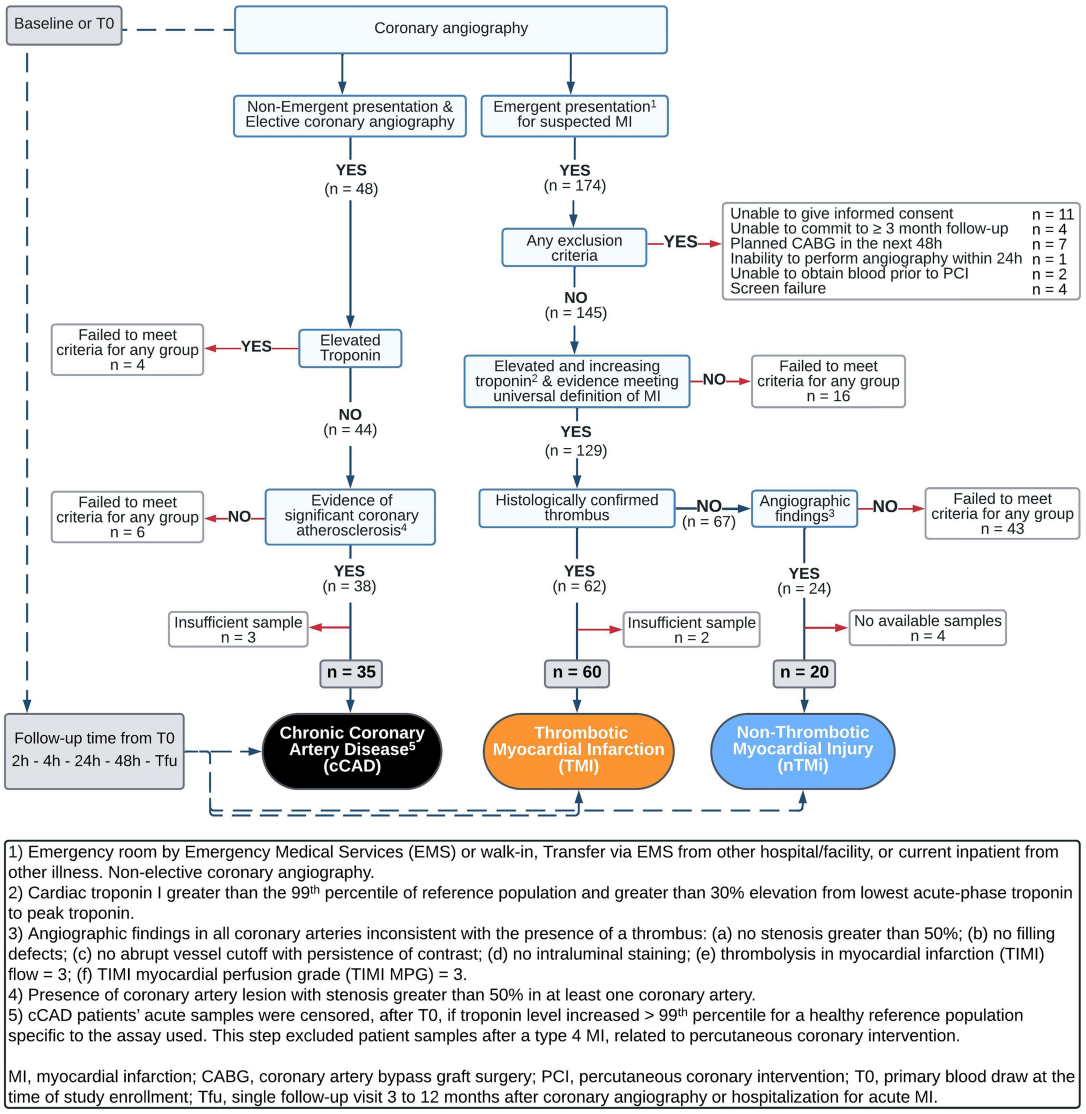
Flowchart for inclusion of patients into cCAD, TMI, and nTMi analytical groups.

The analytical cohort was designed to identify two etiological types of acute myocardial injury (TMI and nTMi) and a non-acute but diseased control (cCAD) (Table 1). The criteria were chosen to maximize analytical group specificity with the expectation of differences in both clinical features and pathobiology (Table 1, Figure 2). The patients themselves served as their own controls, from the time of the acute event (time of invasive coronary angiography for acute myocardial injury or chronic coronary atherosclerosis) to the quiescent state (stable for ≥3 months). This study design allows for the identification of characteristics specific to the acute clinical event (within patients) and differences between event types by comparison between myocardial injury patient types. As compared with TMI, individuals with acute nTMi serve as control for ischemia/necrosis; and individuals with cCAD serve as control for the underlying disease state, atherosclerosis, and diagnostic evaluation (cardiac catheterization).

**Table 1:** Description of study analytical phenotypes (cCAD, TMI, nTMi).

Study group	Presentation	Troponin I levels	Thrombus	Blinded angiographic assessment
cCAD	Elective, planned, outpatient coronary angiography	Baseline troponin I < 0.03 ng/ml	No thrombus aspirated	Stenosis greater than 50% in at least one coronary artery
TMI	Clinical presentation consistent with the Fourth Universal Definition of Myocardial Infarction	Peak troponin I > 0.03 ng/ml and greater than 30% elevation from lowest acute-phase troponin to peak troponin	Thrombus aspirated from the coronary artery and confirmed by blinded pathological assessment	
nTMi	Clinical presentation consistent with the Fourth Universal Definition of Myocardial Infarction	Peak troponin I > 0.03 ng/ml and greater than 30% elevation from lowest acute-phase troponin to peak troponin	No thrombus aspirated	Angiographic findings in all coronary arteries inconsistent with the presence of a thrombus: 1. No stenosis greater than 50% 2. No filling defects 3. No abrupt vessel cutoff with persistence of contrast 4. No intraluminal staining 5. TIMI flow = 3 6. TIMI MPG = 3

#### 2.2.1 Acute thrombotic MI and acute non-thrombotic myocardial injury

Enrollment criteria for acute myocardial injury, which includes TMI and nTMi groups, required that each patient be >18 years of age and scheduled for non-elective coronary angiography within 48 h after admission. Those enrolled in either of the acute phenotypes must have had at least one of the following four criteria: (1) new or presumably new ST-segment depression >0.1 mV; (2) elevated cardiac troponin I >99th percentile for a healthy reference population specific to the assay used and >30% elevation from lowest acute-phase troponin to peak troponin within 24 h of enrollment; (3) ≥1 mm ST-segment elevation in ≥2 contiguous electrocardiogram (ECG) leads; or (4) ≥1 mm ST-segment depression in V1 and V2 (posterior wall infarct) (Table 1) (24). Patients who received fibrinolysis were not eligible. All troponin measurements were performed in a Clinical Laboratory Improvement Amendments certified laboratory.

The criteria for differentiating between TMI and nTMi were based upon those previously proposed by our group (31), as described in Table 1. The definition of TMI included the criteria for acute myocardial injury as well as presence of a histologically confirmed coronary thrombus (by blinded pathological assessment, CVPath Institute, Inc., Gaithersburg, MD, USA) (Table 1). nTMi was defined as meeting the same four criteria for acute myocardial injury as TMI, but with no recovery of a histologically confirmed thrombus, and satisfaction of all of the following six criteria in all coronary vessels via core laboratory blinded angiogram assessment: (1) no stenosis greater than 50%, (2) no filling defects, (3) no abrupt vessel cutoff with persistence of contrast, (4) no intraluminal staining, (5) thrombolysis in myocardial infarction (TIMI) flow = 3, and (6) TIMI myocardial perfusion grade (TIMI MPG) = 3 (24) (Table 1, Figure 2).

#### 2.2.2 Chronic coronary atherosclerotic disease

Patients enrolled in the suspected cCAD group were required to have presented for coronary angiography as an elective procedure, with evidence of significant coronary atherosclerosis with stenosis greater than 50% in at least one coronary vessel; or had a past medical history of atherosclerosis as evidenced by coronary artery bypass graft (CABG), percutaneous coronary intervention (PCI), stroke/ transient ischemic attack (TIA), carotid endarterectomy, peripheral artery bypass procedure, or abdominal aortic aneurysm repair. Additional criteria included normal TIMI flow and TIMI MPG in all vessels via core laboratory blinded angiogram assessment as well as pre-procedure cardiac troponin I <99th percentile for a healthy reference population specific to the assay used. Patients in the suspected cCAD group were excluded on the basis of any one of the following criteria: (1) hospitalization for acute coronary syndrome or clinical instability within 4 weeks prior to planned enrollment; CABG within 1 year prior to planned enrollment; or PCI, stroke, or TIA within 12 weeks prior to planned enrollment; (2) presence of unstable angina or symptoms refractory to maximal medical therapy; (3) presence of significant comorbidities likely to cause death within 2 years; (4) significant active history of substance abuse within 5 years of enrollment; or (5) unable to return to the medical campus for a 3-month stable follow-up (Table 1, Figure 2). Acute samples of patients with cCAD were censored, after T0, if troponin level increased >99th percentile for a healthy reference population specific to the assay used. This step excluded patient samples after a type 4 myocardial infarction, related to percutaneous coronary intervention.

### 2.3 Sample collection and preparation for DSC analysis

Samples from a total of 115 patients (cCAD, TMI, and nTMi) were collected at multiple acute-phase time points (T0, T2, T4, T24, T48) as well as a follow-up (Tfu) when the patient was in a stable state. Enrollment sample collection via an arterial sheath took place at the time of the coronary angiography after a 5–10 ml waste draw. All available follow-up samples (T2, T4, T24, T48, and ≥3 months) were collected from a peripheral vein, utilizing a blood pressure cuff as a gentle tourniquet (maximum pressure of <40 mmHg), after >10 ml of clinical blood collection or waste draw, and into a tube containing ethylenediamine tetraacetic acid (EDTA). Plasma was processed with a standardized protocol 45 min after collection.

Longitudinal plasma samples encompassing multiple time points during the acute time course (T0, T2, T4, T24, and T48) and a stable cardiac state at the 3–12-month follow-up (Tfu) were randomly batched into sets of 14 samples to ensure all sample handling and data collection could be completed within 7 days after sample thawing. We previously validated all aspects of our experimental approach for the analysis of plasma samples (specimen processing and storage, sample preparation and batching for DSC analysis, instrument settings and analysis replicates, and data processing) across thousands of analyses (32). Each batch of samples was prepared for DSC analysis by dialyzing against a standard phosphate buffer (1.7 mM KH_2_PO_4_, 8.3 mM K_2_HPO_4_, 150 mM NaCl, 14.7 mM sodium citrate, pH 7.5) to achieve normalization of buffer conditions for all samples. Specifically, each plasma sample (150–200 µl) was split between two Slide-A-Lyzer MINI dialysis units (MWCO 3500, 0.1 ml; Pierce, Rockford, IL, USA) and dialyzed at 4 °C against 1 L of phosphate buffer for a total dialysis time of 24 h, with buffer changes after 3 h of dialysis, then after two periods of 4 h, followed by a final overnight dialysis period of 14 h. After dialysis, the samples were recovered from dialysis units and filtered to remove particulates using centrifuge tube filters (0.45 μm cellulose acetate; Pall Corporation, New York, NY, USA). The final dialysis buffer was also filtered (0.2 μm polyethersulfone; Pall Corporation) and used for all sample dilutions and as a reference solution for DSC studies. Dialyzed samples were diluted 25-fold with a final dialysis buffer to obtain a suitable protein concentration for DSC analysis (∼ 2 mg/ml). The exact protein concentration of each plasma sample analyzed by DSC was determined using the bicinchoninic acid protein assay kit microplate protocol (Pierce), using absorbance measurements taken with a Tecan Spark plate reader (Tecan US, Research Triangle Park, NC, USA).

### 2.4 TLB profile determination

TLB profiles were generated from DSC data collected with a Nano DSC Autosampler System (TA instruments, New Castle, DE, USA), which was serviced according to the manufacturer's procedures. Interim instrument performance was assessed using the biological standard lysozyme and was within the manufacturer's specifications. The plasma samples and matched final dialysis buffer to load the instrument sample and reference chambers, respectively, were transferred to 96-well plates and loaded into the instrument autosampler maintained at 4 °C until analysis. Sample volumes of 950 μl were required to provide sufficient volume to ensure proper rinsing and filling of the 300 μl thermal sensing area. DSC scans were recorded from 20 to 110 °C at a scan rate of 1 °C/min following a pre-scan equilibration period of 900 s at 20 °C. The instrument was cycled overnight by running multiple water scans followed the next morning by at least three buffer scans to condition the instrument chambers before running the batch of samples. Buffer scans collected at the beginning and end of a sample set and after single or consecutive sample scans were examined to determine acceptable reproducibility and effective rinsing of the instrument chambers. Duplicate DSC scans were obtained for each of the TLB profiles shown in the results to ensure the profile was reproducible. Raw DSC scans were corrected for instrument baseline by subtraction of a suitable buffer reference scan, normalized for sample protein concentrations, and corrected for non-zero sample baselines by application of a linear baseline function using Origin 7 software (OriginLab Corporation, Northampton, MA, USA). TLB profiles were plotted as excess specific heat capacity, C_p_^ex^ (cal/°C.g), vs. temperature (°C) with final analysis performed on a temperature range of 45–90 °C with an interval size of 0.1 °C.

### 2.5 Statistical analysis and data visualization

The baseline characteristics of the patient cohort grouped into three myocardial injury groups were summarized with mean and standard deviation, or median, first quartile (25th percentile) and third quartile (75th percentile), if the distribution showed substantial visual evidence of non-normality or skew. Categorical characteristics were summarized with frequency and proportion within each study group. Since the analytical cohorts were different by design, statistical testing of differences was not performed.

**Figure 3:**
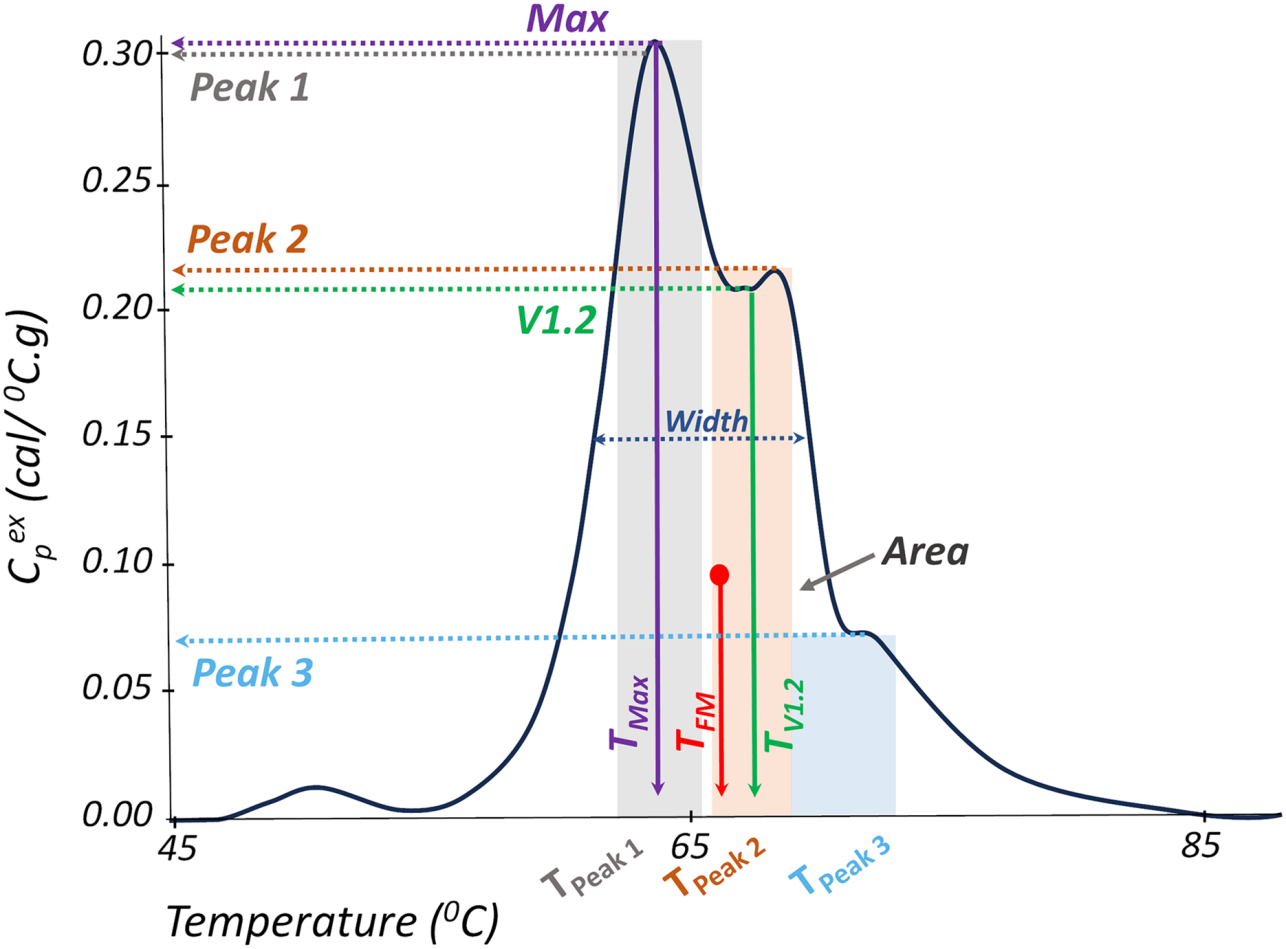
Representation of TLB metrics evaluated in this study. TLB profile width at half height (*Width*); total area of the TLB profile (*Area*), maximum profile amplitude (*Max*); temperature of the maximum profile amplitude (*T_Max_*); first moment temperature (*T_FM_*); peak amplitudes corresponding to the temperature regions 60–66 °C (*Peak 1*), 67–73 °C (*Peak 2*), and 73–81 °C (*Peak 3*); temperature of *Peak 1* (*T_Peak 1_*), *Peak 2* (*T_Peak 2_*), and *Peak 3* (*T_Peak 3_*); the minimum (valley) between *Peak 1* and *Peak 2* (*V1.2*); and temperature of *V1.2* (*T_V1.2_*).

A panel of 19 TLB metrics (Figure 3) was utilized to characterize all TLB profiles at baseline (T0) and quiescent phase (Tfu) time points. The changes in all 19 TLB metrics within patients, between the quiescent state and the acute phase presentation (ΔTfu − T0), were also evaluated. The 19 TLB metrics were as follows: peak amplitudes corresponding to the temperature region 60–66 °C (*Peak 1*), 67–73 °C (*Peak 2*), and 73–81 °C (*Peak 3*); the temperature of *Peak 1* (*T_Peak 1_*), *Peak 2* (*T_Peak 2_*), and *Peak 3* (*T_Peak 3_*); the ratio of *Peak 1* and *Peak 2* amplitudes (*Peak 1/Peak 2*); the ratio of *Peak 1* and *Peak 3* amplitudes (*Peak 1/Peak 3*); the ratio of *Peak 2* and *Peak 3* amplitudes (*Peak 2/Peak 3*); the minimum (valley) between *Peak 1* and *Peak 2* (*V1.2*); temperature of *V1.2* (*T_V1.2_*); the ratio of *V1.2* and *Peak 1* (*V1.2/Peak 1*); the ratio of *V1.2* and *Peak 2* (*V1.2/Peak 2*); the ratio of *V1.2* and *Peak 3* (*V1.2/Peak 3*); the maximum TLB profile amplitude (*Max*); the temperature of *Max* (*T_Max_*); the first moment temperature (*T_FM_*); TLB profile width at half height (*Width*); and the total area of the TLB profile (*Area*). Peak identification was based on a predetermined temperature range of three major transition ranges typically observed in TLB profiles—a major transition (*Peak 1*) in the range 60–66 °C, a smaller transition (*Peak 2*) in the range 67–73 °C, and a shoulder transition (*Peak 3*) in the range 73–81 °C, within which the maximum amplitude was recognized as the peak position (12, 28, 33, 34). The valleys were determined by locating the lowest amplitude between any two given peaks. All TLB metrics were derived using the *tlbparam* R package available at http://www.github.com/BuscagliaR/tlbparam.

Non-parametric testing was used to alleviate failed normality assumptions of linear models. The Kruskal–Wallis test with correction for multiple comparisons was utilized to determine if there was evidence of differences in TLB metric distribution across the patient groups. Statistical significance indicates that the values of a TLB metric are consistently larger/smaller in at least one group, suggesting a systematic difference in the metric's distribution by patient group. To explore pairwise differences between groups, the Wilcoxon signed-rank test was utilized, with group comparisons visualized by box and whisker plots.

Using the full TLB profile, the ability to differentiate myocardial injury type at baseline (T0) was investigated using unsupervised methodologies that required no *a priori* assumption regarding patient status via clustering of patient TLB profiles related to an acute myocardial injury event, followed by an assessment of cluster purity and characteristics. Importantly, the clustering process exclusively utilized only TLB profiles, remaining unaffected by additional clinical factors or patient information, such as myocardial injury phenotype. The numbers of clusters were assessed based on within-sum-of-squares and silhouette analysis, providing independent measures of the optimal number of clusters (Supplementary Figure S1). Final unsupervised clusters were chosen based on cluster statistics and cluster purity.

All statistical conclusions were based on a 5% significance level. The analyses reported in the current work were conducted using the statistical programming language R and the following packages: *dplyr*, *tidyr*, *ggplot2*, *stat*, and *factoextra* (35–37).

## 3 Results

The baseline cohort characteristics for the three patient groups analyzed, acute TMI (*n* = 60), acute nTMi (*n* = 20), and cCAD (*n* = 35), are displayed in Table 2. Patients with TMI were younger, predominantly male, with more being smokers as compared with patients with cCAD or nTMi. Patients with cCAD were more likely to be White, former smokers, dyslipidemic, diabetic, hypertensive, and had a prior history of atherosclerosis, heart failure, and lower platelet counts as compared with patients with TMI or nTMi. At baseline, ST elevation was observed in 78% of the patients with TMI, and 30% of the patients with nTMi (Table 2). Differences in history of atherosclerosis, coronary stenosis ≥75%, median troponin at enrollment, and peak troponin varied as expected based on the criteria used to define the study cohorts. From baseline to T48, 88% of the patients with TMI had a peak troponin >100 times the URL. Most of the patients with nTMi fell within the range of 10–100 times the URL, whereas patients with cCAD had peak troponin below the threshold of 1 URL.

**Table 2:** Characteristics of the patients in the study cohort.

Variable	cCAD (*n* = 35)	TMI (*n* = 60)	nTMi (*n* = 20)
Age (mean ± SD), years	64.61 ± 10.19	56.87 ± 10.98	57.67 ± 14.46
Male Sex, *n* (%)	29 (82.9)	44 (73.3)	7 (35.0)
Race, *n* (%)			
Black	3 (8.6)	6 (10.0)	6 (30.0)
White	32 (91.4)	53 (88.3)	13 (65.0)
Other	0 (0.0)	1 (1.7)	1 (5.0)
Smoking history, *n* (%)			
Current	5 (14.3)	33 (55.0)	7 (35.0)
Former	17 (48.6)	14 (23.3)	8 (40.0)
Never	13 (37.1)	13 (21.7)	5 (25.0)
Alcohol history, *n* (%)			
Current	11 (31.4)	26 (43.3)	5 (25.0)
Former	11 (31.4)	15 (25.0)	4 (20.0)
Never	13 (37.1)	19 (31.7)	11 (55.0)
Dyslipidemia, *n* (%)			
Yes	31 (88.6)	33 (55.0)	8 (40.0)
No	3 (8.6)	27 (45.0)	12 (60.0)
Diabetes, *n* (%)	12 (34.3)	16 (26.7)	6 (30.0)
Hypertension, *n* (%)	30 (85.7)	31 (51.7)	13 (65.0)
History of atherosclerosis^a^, *n* (%)			
Yes	29 (82.9)	11 (18.3)	4 (20.0)
No	5 (14.3)	49 (81.7)	16 (80.0)
History of congestive heart failure, *n* (%)			
Yes	6 (17.1)	3 (5.0)	5 (25.0)
No	29 (82.9)	57 (95.0)	15 (75.0)
History of chronic renal failure, *n* (%)			
Yes	3 (8.6)	1 (1.7)	2 (10.0)
No	32 (91.4)	59 (98.3)	17 (85.0)
^b^Heart rate (mean ± SD), bpm	68.83 ± 14.41	80.05 ± 20.13	81.30 ± 17.44
^b^SBP (mean ± SD) mm Hg	144.74 ± 21.10	135.35 ± 24.60	132.30 ± 25.43
^b^DBP (mean ± SD) mm Hg	82.54 ± 13.85	86.13 ± 16.05	80.50 ± 18.87
^b^Mean arterial pressure (mean ± SD) mm Hg	103.28 ± 14.23	102.54 ± 18.16	97.77 ± 19.75
Creatinine at enrollment^c^ [median (Q1, Q3)], mg/dl	0.99 (0.83, 1.12)	0.98 (0.87, 1.07)	0.85 (0.68, 1.08)
Platelets (mean ± SD), ×10^9^/L	207.23 ± 66.14	255.52 ± 65.68	256.50 ± 69.87
Stenosis ≥ 75%, *n* (%)	25 (71.4)	59 (98.3)	0 (0.0)
^b^Troponin at enrollment^d^ [median (Q1, Q3)], ng/ml	0.01 (0.01, 0.01)	0.20 (0.04, 1.04)	1.66 (0.97, 3.35)
^e^Peak troponin from T0 to T48 relative to the URL^f^, *n* (%)			
<1 URL	25 (73.6)	0 (0.0)	0 (0.0)
1 to <10 URL	3 (8.8)	1 (1.7)	3 (15.0)
10 to <100 URL	5 (14.7)	6 (10.0)	12 (60.0)
≥100 URL	1 (2.9)	53 (88.3)	5 (25.0)
^b^ST elevation on ECG at presentation, *n* (%)			
Yes	0 (0.0)	47 (78.3)	6 (30.0)
No	34 (100.0)	12 (20.0)	13 (65.0)

Categorical variables are summarized with frequency and percentage within group. Continuous variables are summarized with mean ± standard deviation or median (25th percentile, 75th percentile).

BP, blood pressure; Q1, first quartile (25th percentile); Q3, third quartile (75th percentile); SBP, systolic blood pressure; DBP, diastolic blood pressure.

^a^ History of Atherosclerosis includes previous MI, CAD, PCI, or CABG.

^b^ At time of presentation.

^c^ Three values were unavailable in TMI, two in nTMi, and one in cCAD.

^d^ One value was unavailable in TMI.

^e^ Troponin URL of 0.040 ng/ml (Beckman Access assay).

^f^ Per protocol, one cCAD patient had T2 to T48 samples excluded after a type 4 MI related to percutaneous coronary intervention.

### 3.1 Baseline TLB profiles

**Figure 4:**
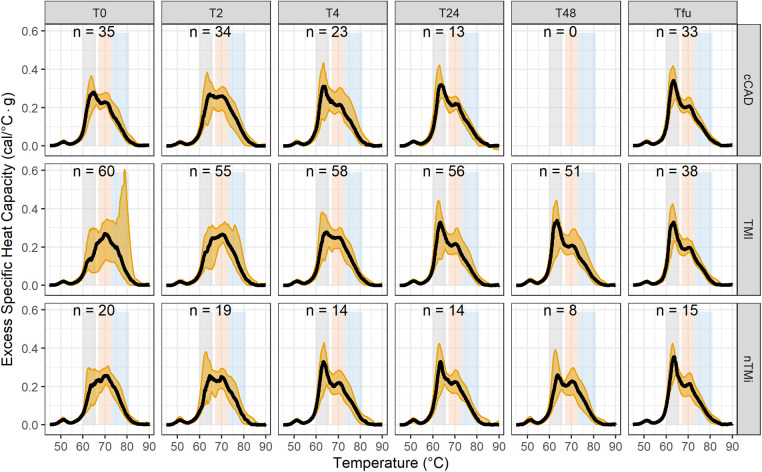
Time-course evaluation of myocardial injury phenotypes presented as median TLB profile (black) and 95% quantile interval (gold shading). Note: TLB profiles of blood plasma samples are commonly characterized by three main transitions: a major transition (*Peak 1*, 60–66 °C), with a smaller transition (*Peak 2*, 67–73 °C), and a shoulder transition (*Peak 3*, 73–81 °C), as represented by the gray, orange, and blue bands, respectively.

By visual inspection, median TLB profiles demonstrate regions of differentiation among all three patient groups at baseline (T0) (Figure 4). At the time of an acute event (T0), both TMI and nTMi had a lower Peak 1 as compared with cCAD, and TMI had a higher Peak 3 as compared with both cCAD and nTMi.

**Figure 5:**
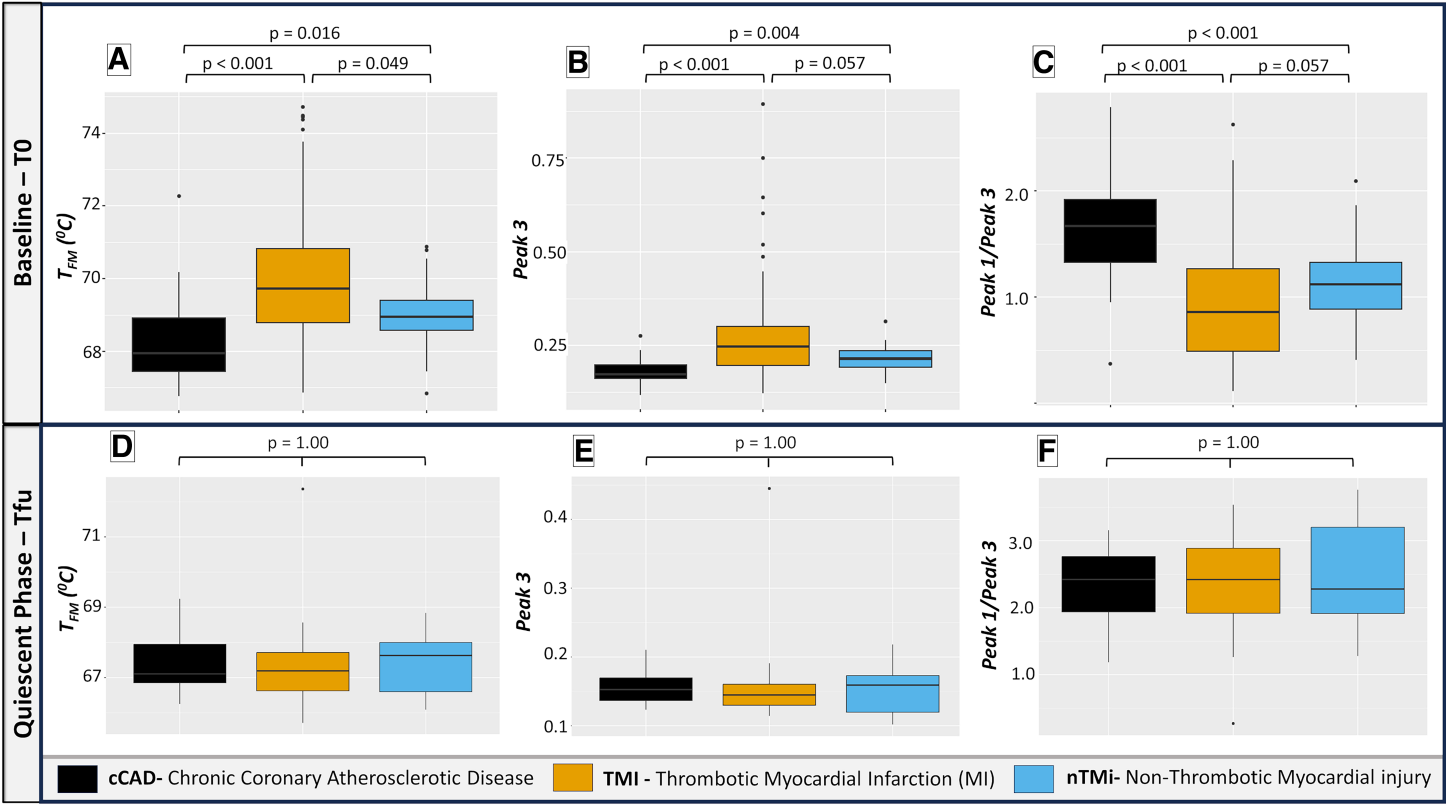
Box plots illustrating selected TLB profile metrics at baseline (T0) and quiescent phase (Tfu) comparing cCAD, TMI, and nTMi. Among the 19 evaluated TLB metrics, *T_FM_*, *Peak 3,* and *Peak 1/Peak 3* emerge as the most clinically significant, with the potential to differentiate between TMI and nTMi. Pairwise Wilcoxon signed-rank tests demonstrate distinct TLB differentiation at T0 among all three groups in (**A**) while (**B**,**C**) differentiate acute myocardial injury (TMI and nTMi) from cCAD at T0. (**D–F**) show a similar distribution of TLB metric values among all groups at Tfu. Note: *T_FM_***_:_** first moment temperature; *Peak 3*: peak amplitude corresponding to the temperature region 73–81 °C; *Peak 1/Peak 3*: ratio of *Peak 1* and *Peak 3* amplitudes.

Among the 19 TLB metrics, 13 were different between at least two of the patient groups at T0 (Table 3). Differences in 10 out of the 13 metrics, *Peak 1*, *Peak 3*, *T_Peak 2_*, *Peak 1*/*Peak 2*, *Peak 1/Peak 3*, *Peak 2/Peak 3*, *V1.2/Peak 2*, *V1.2/Peak 3*, *T_FM_*, and *T_Max_*, were observed between patients with TMI vs. those with cCAD or nTMi vs. patients with cCAD at T0 (Figure 5, Supplementary Figure S2). Differences in three out of 13 metrics were observed between patients with TMI and those with cCAD at T0. Importantly, one metric, *T_FM_*, showed significant differences between all three groups and was able to distinguish TMI from cCAD, nTMi from cCAD, as well as TMI from nTMi at T0 (Figure 5, Supplementary Tables S1, S2). The TLB profiles grouped by clinical phenotype at T0 are provided in Supplementary Figure S3.

**Table 3:** Summary of the analysis assessing differences in distributions of the values of 19 TLB metrics across myocardial injury patient groups.

TLB metric	Baseline (T0)		Quiescent phase (Tfu)		ΔTfu − T0	
	Unadjusted *p*-value	Adjusted *p*-value	Unadjusted *p*-value	Adjusted *p*-value	Unadjusted *p*-value	Adjusted *p*-value
*Peak 1*	**<0.001**	**<0.001**	0.549	1.000	**0**.**001**	**0**.**019**
*Peak 2*	**0.004**	**0.028**	0.365	1.000	**0**.**016**	0.202
*Peak 3*	**<0.001**	**<0.001**	0.372	1.000	**< 0.001**	**< 0.001**
*Peak 1/Peak 2*	**<0.001**	**<0.001**	0.926	1.000	0.215	1.000
*Peak 1/Peak 3*	**<0.001**	**<0.001**	0.900	1.000	**0**.**036**	0.417
*Peak 2/Peak 3*	**<0.001**	**<0.001**	0.559	1.000	0.300	1.000
*V1.2*	**0.002**	**0.022**	0.127	1.000	0.224	1.000
*T_V1.2_*	**<0.001**	**<0.000**	0.033	0.600	**0**.**001**	**0**.**012**
*V1.2/Peak 1*	**0.042**	0.209	0.709	1.000	0.357	1.000
*V1.2/Peak 2*	**<0.001**	**<0.001**	0.330	1.000	0.419	1.000
*V1.2/Peak 3*	**<0.001**	**<0.001**	0.529	1.000	0.297	1.000
*Max*	**0.011**	0.079	0.785	1.000	0.113	1.000
*T_Peak 1_*	0.341	0.969	0.106	1.000	0.300	1.000
*T_Peak 2_*	**<0.001**	**0.002**	0.200	1.000	**0**.**035**	0.417
*T_Peak 3_*	0.196	0.784	0.532	1.000	0.176	1.000
*T_Max_*	**<0.001**	**<0.001**	0.117	1.000	**<0.001**	**<0.001**
*T_FM_*	**<0.001**	**<0.001**	0.541	1.000	**<0.000**	**<0.001**
*Width*	**0.014**	0.086	0.925	1.000	0.209	1.000
*Area*	0.323	0.969	0.007	0.133	**0**.**007**	0.101

Peak amplitudes corresponding to the temperature region 60–66 °C (*Peak 1*), 67–73 °C (*Peak 2*), and 73–81 °C (*Peak 3*); the ratio of *Peak 1* and *Peak 2* amplitudes (*Peak 1/Peak 2*); the ratio of *Peak 1* and *Peak 3* amplitudes (*Peak 1/Peak 3*); the ratio of *Peak 2* and *Peak 3* amplitudes (*Peak 2/Peak 3*); the minimum (valley) between *Peak 1* and *Peak 2* (*V1.2*); temperature of *V1.2* (*T*_*V1.2*_); the ratio of *V1.2* and *Peak 1* (*V1.2/Peak 1*); the ratio of *V1.2* and *Peak 2* (*V1.2/Peak 2*); the ratio of *V1.2* and *Peak 3* (*V1.2/Peak 3*); the maximum profile amplitude (*Max*); the temperature of *Peak 1* (*T*_*Peak 1*_), *Peak 2* (*T*_*Peak 2*_), and *Peak 3* (*T*_*Peak 3*_); the temperature of maximum profile amplitude (*T*_*Max*_); the first moment temperature (*T*_*FM*_); profile width at half height (*Width*); and the total area of the TLB profile (*Area*).

Statistically significant differences (*p* < 0.05) are shown in bold indicating TLB metrics that show differences in at least one patient group and thus have the ability to differentiate myocardial injury types at baseline (T0), quiescent phase (Tfu), and the difference between Tfu and T0 (ΔTfu − T0).

### 3.2 Quiescent phase TLB profiles

By visual inspection, median TLB profiles are similar for all three patient types (cCAD, TMI, nTMi) at the quiescent phase (Tfu) (Figure 4). TLB profiles at Tfu, following the resolution of the acute myocardial injury event, have a large *Peak 1* amplitude, a lower *Peak 2* amplitude, and a small *Peak 3* shoulder. Patients with cCAD maintain the least profile variability across the time course, in contrast to both those with TMI or nTMi. In addition, all 19 TLB metrics were found to have no statistical differences in distribution across the myocardial injury groups at Tfu (Figure 4, Table 3).

### 3.3 Time-course TLB profiles: contrasting baseline and quiescent phase

Changes between baseline and quiescent phase, within the patient groups, are least pronounced in the patients with cCAD as compared with those with TMI or nTMi. An acute disease state results in a diminished *Peak 1* and more prominent *Peaks 2* and *3*, compared with a dominant *Peak 1* TLB signature for the quiescent state (Figure 4). The time course represents an enriched view for tracking changes in myocardial injury, with all patient groups demonstrating recovery of the dominant *Peak 1* TLB signature at the quiescent time point. The TLB of patients with TMI demonstrated a highly diminished *Peak 1* and elevated *Peaks 2* and *3* at T0 as compared with the group of patients with non-acute diseased cCAD that received the same invasive diagnostic angiogram at T0 but were not having an acute myocardial event. For patients with nTMi, the TLB profile at T0 was also distinct from those with cCAD, with a diminished *Peak 1* and elevated *Peak 2*, and was further distinct from those with TMI with distinctive time-dependent changes of the TLB profile. The time course for both TMI and nTMi demonstrated a gradual change in the median TLB profile to the quiescent state TLB profile, but with differences in the dynamics of the recovery of the dominant *Peak 1* TLB signature across the time course.

**Figure 6:**
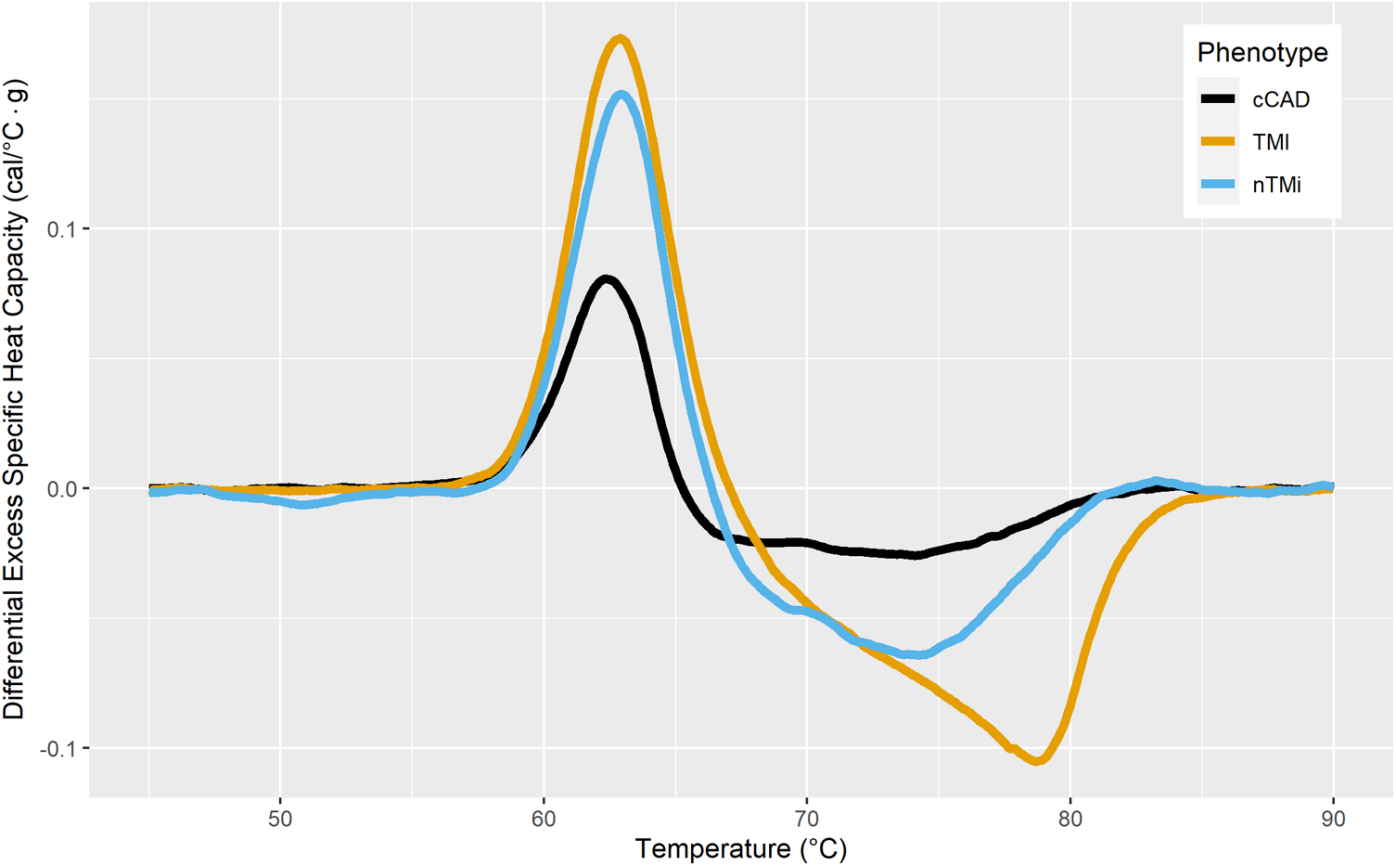
Mean difference TLB profiles between stable cardiac state at the 3–12-month follow-up (Tfu) and baseline (T0) colored by myocardial injury phenotype.

Figure 6 presents the mean TLB difference profile observed for the differences between Tfu and T0 TLB profiles. cCAD showed a smaller mean change between T0 and Tfu with a lower amplitude of *Peak 1* at T0, with minimal change in the *Peak 2* and *Peak 3* regions. TMI showed the most extreme mean differences, with a large positive change in *Peak 1* and a large negative change in *Peak 3* between T0 and Tfu time points. This reflects a change from a depressed *Peak 1* and large 80 °C peak at T0, to a TLB signature with a prominent *Peak 1* and no significant signal at 80 °C at Tfu. nTMi shows diversity from these groups in its difference in the region between 68 and 75 °C, while having a slightly smaller change in *Peak 1* compared with TMI. The TLB difference profiles for all patients having paired TLB profiles (Tfu − T0) are presented in Supplementary Figure S4.

Among the 19 TLB metrics assessed across the three study groups, five metrics showed distinct changes from T0 to Tfu, including *T_FM_*, *T_Max_*, *Peak 1*, *Peak 3*, and *T_V1.2_* (Supplementary Tables S1, S2).

**Figure 7:**
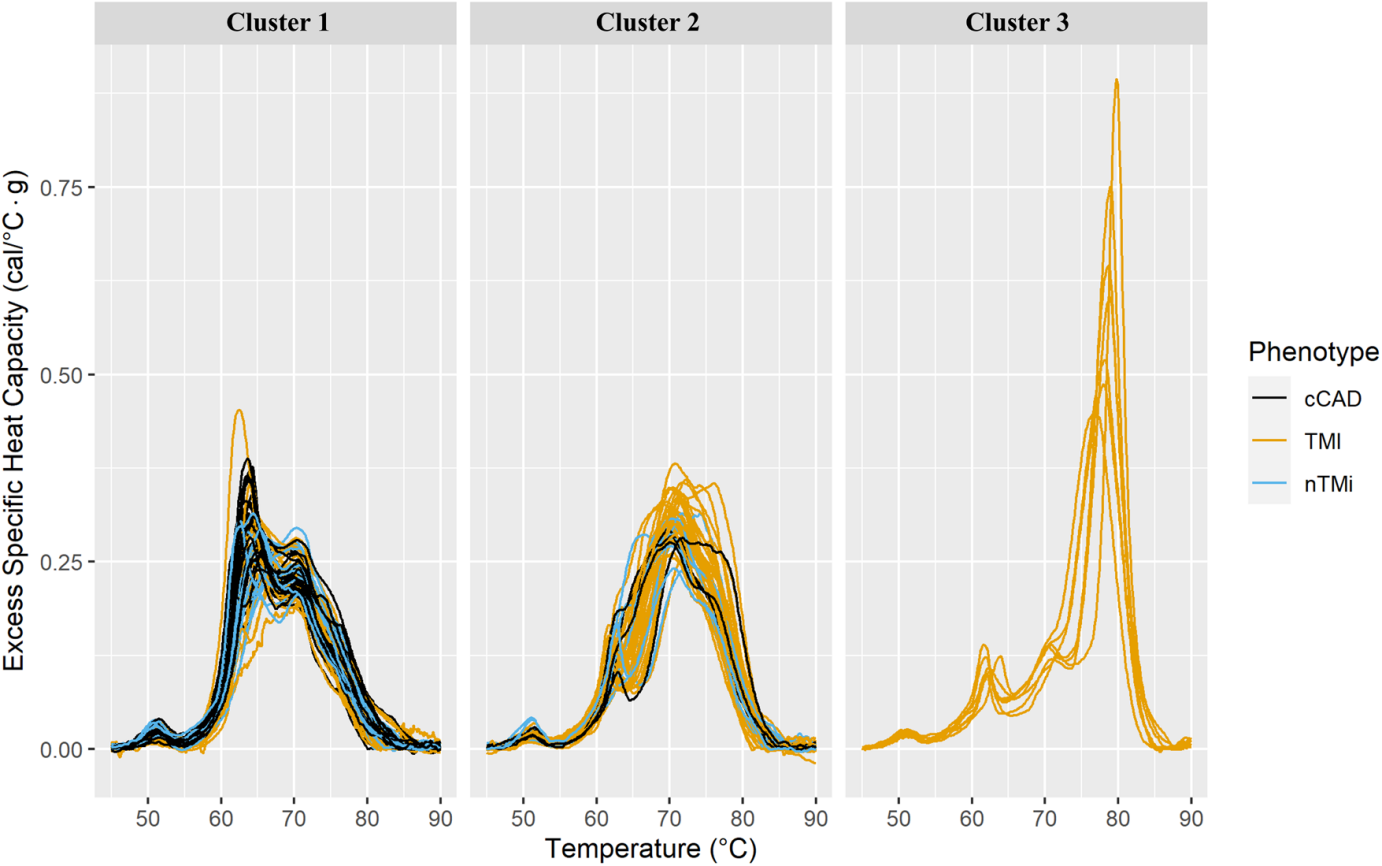
Faceted TLB profile clusters (arbitrarily labeled clusters 1, 2, and 3) for three k-mean centers at baseline (T0). The columns correspond to the unsupervised cluster label. All samples within a given cluster are shown colored by clinical phenotype. The phenotypes and corresponding colors are provided in the legend.

Unsupervised clustering was employed to identify unique groupings of TLB profiles. Through the use of k-means clustering, it was determined that an optimal cluster size included *k* = 3 cluster centers, with the assessment across cluster sizes provided in Supplementary Figure S1. The finalized clusters are presented in Figure 7 colored by clinical phenotype, with phenotype purity presented in Table 4. Cluster 1 predominantly comprises patients with cCAD (47%) but includes those with TMI (34.8%) and nTMi (18.2%) as well. Cluster 2 contains a mix of all patient groups but is predominantly TMI (>70%). It is characterized by a loss of *Peak 1* definition, and a tendency to shift toward higher peak temperatures. Cluster 3 shows a distinct pattern, unlike the other clusters, having small *Peak 1* and *Peak 2* amplitudes, with a dominant and clearly defined 80 °C peak rarely observed at such a large amplitude. Cluster 3 only contains patients with TMI, with a TLB profile distinct from that observed within the Cluster 2 patients with TMI who show substantial shifting of the TLB profile without the development of the 80 °C peak.

**Table 4:** Cluster purities for three k-mean centers at baseline (T0).

Phenotype	Cluster 1	Cluster 2	Cluster 3
cCAD (*n* = 35)	31 (47.0%)	4 (9.5%)	0 (0.0%)
TMI (*n* = 60)	23 (34.8%)	30 (71.5%)	7 (100.0%)
nTMi (*n* = 20)	12 (18.2%)	8 (19.0%)	0 (0.0%)
Total samples in cluster	66	42	7

Each column provides the number of samples and proportion of the cluster represented by the three clinical phenotypes. The total number of samples within each cluster is also provided.

## 4 Discussion

This study demonstrates the potential utility of TLB as a serological assay for distinguishing and characterizing myocardial injury events. The key findings include the following: (1) distinctive patterns in TLB profile among the three clinically relevant myocardial injury groups (cCAD, TMI, nTMi) at the time of acute event/evaluation; (2) the TLB profile was substantially altered for TMI and nTMi at the time of the acute event compared with the quiescent phase; (3) relatively less pronounced change in TLB profile from the time of the acute evaluation to the quiescent phase for the cCAD group as compared with the TMI and nTMi; (4) at quiescent phase, TLB profiles for all three patient groups were similar; (5) TLB characteristics can differentiate acute events (TMI/nTMi) from cCAD; (6) an identifiable TLB signature unique to the TMI group is observed in unsupervised clustering and with one TLB metric. Understanding the pathobiology of acute myocardial injury phenotypes has the potential to foster the development of innovative diagnostic, prognostic, preventative, and therapeutic modalities specific to etiologically unique and clinically important disease phenotypes.

The distinct TLB profiles observed during event presentation among myocardial injury groups underscores the diverse pathobiology within the three patient groups (cCAD, TMI, nTMi). However, the areas of similarities in TLB profiles between TMI and nTMi may indicate underlying TLB-captured mechanisms of shared resultant myocardial injuries. The current diagnostic criteria for acute MI lack the ability to delineate the cause of MI in a clinically actionable manner, resulting in non-specific treatments and missed opportunities to intervene prior to irreversible myocardial necrosis, even with the inclusion of high-sensitivity cardiac troponin (hs-cTn) (38).

The current study identified one TLB metric (*T_FM_*) that distinguished between TMI, nTMi, and cCAD. This TLB metric may be reflective of the specific pathobiological state, including plaque disruption and atherothrombosis, that is distinct from the shared biology of myocardial injury and chronic atherosclerosis. These differences may be further investigated by combining proteomic, lipidomic, or metabolomic data with the TLB profile signatures (18). In a prior investigation conducted by our group, we characterized 1,032 plasma metabolites by mass spectrometry in a subset of the patients with TMI, nTMi, and cCAD. We identified a 17-metabolite model that was able to uniquely identify TMI, nTMi, and cCAD at the time of acute myocardial injury event or stable disease evaluation (30). The robust application of TLB in conjunction with biochemical data has identified biochemical mechanisms to better understand thermal stability shifts in major plasma proteins in multiple myeloma phenotypes (15). Similarly, the TLB approach led to a TLB-based prognostic classification for early renal function decline in type 1 diabetes (27) and differentiation of premalignant from benign pancreatic cysts (39). In addition, several proof-of-principle studies demonstrated distinctive TLB signatures for patients with glioblastoma (21), melanoma (32), and psoriasis (25), indicating the potential utility of TLB as a minimally invasive monitoring tool for such diseases. Interestingly, Velazquez-Campoy et al. (32) observed a similar TLB profile for melanoma patients with no evidence of disease and healthy controls, demonstrating TLB as a useful tool for monitoring disease remission, and response to treatment. Although healthy controls were not evaluated in this study, the time-course TLB profiles for our cCAD control group and quiescent stage follow-up (Tfu) for all three patient groups were similar to the dominant Peak 1 TLB signature for quiescent/control groups reported from other previous TLB studies (12, 14, 15, 32).

Our study was limited by sample size but mitigated by our unique study design that used patients as their own controls to identify change from the time of an acute event to a quiescent state, and compared this with a control group of patients with cCAD with the same underlying disease state (atherosclerosis) who were undergoing the same diagnostic procedure (invasive angiography). A larger sample size would allow for more in-depth analysis of TLB profiles related to clinical factors at the time of acute myocardial injury. Another limitation is that the differentiation of patients with myocardial injuries might be related to the magnitude of myocardial damage. Further studies are warranted to better understand the association between TLB signatures and patients with myocardial injuries, irrespective of the extent of myocardial injuries indicated by peak troponin levels. An additional limitation was that the clustering results of TLB profiles showed impurities in the differentiation of cCAD and nTMi from the pervasive TMI phenotype; however, larger sample numbers could allow for additional machine learning and statistical approaches that improve diagnostic performance. Findings from this study warrant further investigation in larger cohorts given the potential for TLB, and the combination of TLB with additional omics datasets, to provide complementary diagnostic approaches and new insights into the biological underpinnings of distinct, clinically relevant myocardial injury events.

## 5 Conclusion

This study represents the first report of the application of TLB as a sensitive and data-rich technique to be explored in the identification and differentiation of acute myocardial injury etiological subtypes.

## Data Availability

The datasets presented in this article are not readily available because they are being utilized to develop clinically available diagnostics tests/diagnostic aids for the identification and classification of acute myocardial injury. Commercial partnership is fostering this research. All data requests will be individually reviewed and honored in a fashion that is specific to the question being asked without divulging trade secrets. Requests to access the datasets should be directed to the corresponding authors at andrew.defilippis@vumc.org or nichola.garbett@louisville.edu.
