# Supplementary material for: Characterization of myocardial injury phenotype by thermal liquid biopsy
Source: Front Cardiovasc Med. 2024 Apr 4;11:1342255. doi: 10.3389/fcvm.2024.1342255 (PMC11024444; doi:10.3389/fcvm.2024.1342255)
Supplement: Supplementary file 1 [file Datasheet1.pdf]

Supplementary Material

Supplemental Table 1. Summary of the analysis to assess pairwise differences in distributions across time course of 13 TLB metrics that were significant from the Kruskal-Wallis test. Statistically significant differences ($p < 0.05$) are shown in bold indicating TLB metrics that have the ability to differentiate myocardial injury types at baseline (T0), and the difference between Tfu and T0 (Δ Tfu – T0). None of the 19 TLB metrics are statistically significant for Tfu, data not presented in this table.

**TLB Metric**	***Baseline (T0)***			***Δ Tfu – T0***		
	**TMI vs cCAD *p-value***	**nTMi vs cCAD *p-value***	**nTMi vs TMI *p-value***	**TMI vs cCAD *p-value***	**nTMi vs cCAD *p-value***	**nTMi vs TMI *p-value***
Peak 1	**< 0.001**	**0.002**	0.092	**0.001**	0.056	0.703
Peak 2	**0.004**	0.101	0.250	NA	NA	NA
Peak 3	**< 0.001**	**0.004**	0.057	**< 0.001**	**0.007**	0.074
Peak 1/Peak 2	**< 0.001**	**0.001**	0.113	NA	NA	NA
Peak 1/Peak 3	**< 0.001**	**0.001**	0.057	NA	NA	NA
Peak 2/Peak 3	**< 0.001**	**< 0.001**	0.516	NA	NA	NA
V1.2	**0.002**	0.288	0.288	NA	NA	NA
$T_{V1.2}$	**< 0.001**	0.071	0.071	**0.005**	**0.001**	0.757
V1.2/Peak 2	**< 0.001**	**0.001**	0.148	NA	NA	NA
V1.2/Peak 3	**< 0.001**	**< 0.001**	0.153	NA	NA	NA
$T_{Peak\ 2}$	**0.001**	**0.001**	0.767	NA	NA	NA
T_{Max}	**< 0.001**	**0.003**	0.443	**< 0.001**	**0.033**	0.091
T_{FM}	**< 0.001**	**0.016**	**0.049**	**< 0.001**	0.076	0.076

Note: Peak amplitudes corresponding to the temperature region 60-67 °C (*Peak 1*), 67-73 °C (*Peak 2*), and 73-81 °C (*Peak 3*); the ratio of *Peak 1* and *Peak 2* amplitudes (*Peak 1/Peak 2*); the ratio of *Peak 1* and *Peak 3* amplitudes (*Peak 1/Peak 3*); the ratio of *Peak 2* and *Peak 3* amplitudes (*Peak 2/Peak 3*); the minimum (valley) between *Peak 1* and *Peak 2* (*V1.2*); temperature of *V1.2* ($T_{V1.2}$); the ratio of *V1.2* and *Peak 2* (*V1.2/Peak 2*); the ratio of *V1.2* and *Peak 3* (*V1.2/Peak 3*); the temperature of *Peak 2* ($T_{Peak\ 2}$); the temperature of maximum profile amplitude (T_{Max}); and the first moment temperature (T_{FM}).

NA: not applicable. TLB metrics marked as NA did not pass the intial nonparametric ANOVA test and thus were consequently excluded from the pairwise Wilcoxon signed-rank test.

Supplemental Table 2. Summary of the analysis to assess pairwise differences in distributions across myocardial injury groups of 13 TLB metrics that were significant from the Kruskal-Wallis test. Statistically significant differences ($p<0.05$) are shown in bold indicating TLB metrics that have the ability to differentiate myocardial injury types at baseline (T0), and the difference between Tfu and T0 (Δ Tfu – T0). None of the 19 TLB metrics are statistically significant for Tfu, data not presented in this table.

TLB Metric	TMI vs cCAD		nTMi vs cCAD		nTMi vs TMI	
	Baseline (T0) p-value	***Δ Tfu – T0 p-value***	***Baseline (T0) p-value***	***Δ Tfu – T0 p-value***	***Baseline (T0) p-value***	***Δ Tfu – T0 p-value***
Peak 1	**< 0.000**	**0.001**	**0.002**	0.056	0.092	0.703
Peak 2	**0.004**	NA	0.101	NA	0.250	NA
Peak 3	**< 0.001**	**< 0.001**	**0.004**	**0.007**	0.057	0.074
Peak 1/Peak 2	**< 0.001**	NA	**0.001**	NA	0.113	NA
Peak 1/Peak 3	**< 0.001**	NA	**0.001**	NA	0.057	NA
Peak 2/Peak 3	**< 0.001**	NA	**< 0.001**	NA	0.516	NA
V1.2	**0.002**	NA	0.288	NA	0.288	NA
$T_{V1.2}$	**< 0.001**	**0.005**	0.071	**0.001**	0.071	0.757
V1.2/Peak 2	**< 0.001**	NA	**0.001**	NA	0.148	NA
V1.2/Peak 3	**< 0.001**	NA	**<0.001**	NA	0.153	NA
$T_{Peak\ 2}$	**0.001**	NA	**0.001**	NA	0.767	NA
T_{Max}	**< 0.001**	**< 0.001**	**0.003**	**0.033**	0.443	0.091
T_{FM}	**< 0.001**	**< 0.001**	**0.016**	0.076	**0.049**	0.076

Note: Peak amplitudes corresponding to the temperature region 60-67 °C (*Peak 1*), 67-73 °C (*Peak 2*), and 73-81 °C (*Peak 3*); the ratio of *Peak 1* and *Peak 2* amplitudes (*Peak 1/Peak 2*); the ratio of *Peak 1* and *Peak 3* amplitudes (*Peak 1/Peak 3*); the ratio of *Peak 2* and *Peak 3* amplitudes (*Peak 2/Peak 3*); the minimum (valley) between *Peak 1* and *Peak 2* (*V1.2*); temperature of *V1.2* ($T_{V1.2}$); the ratio of *V1.2* and *Peak 2* (*V1.2/Peak 2*); the ratio of *V1.2* and *Peak 3* (*V1.2/Peak 3*); the temperature of *Peak 2* ($T_{Peak\ 2}$); the temperature of maximum profile amplitude (T_{Max}); and the first moment temperature (T_{FM}).

NA: not applicable. TLB metrics marked as NA did not pass the intial nonparametric ANOVA test and thus were consequently excluded from the pairwise Wilcoxon signed-rank test.

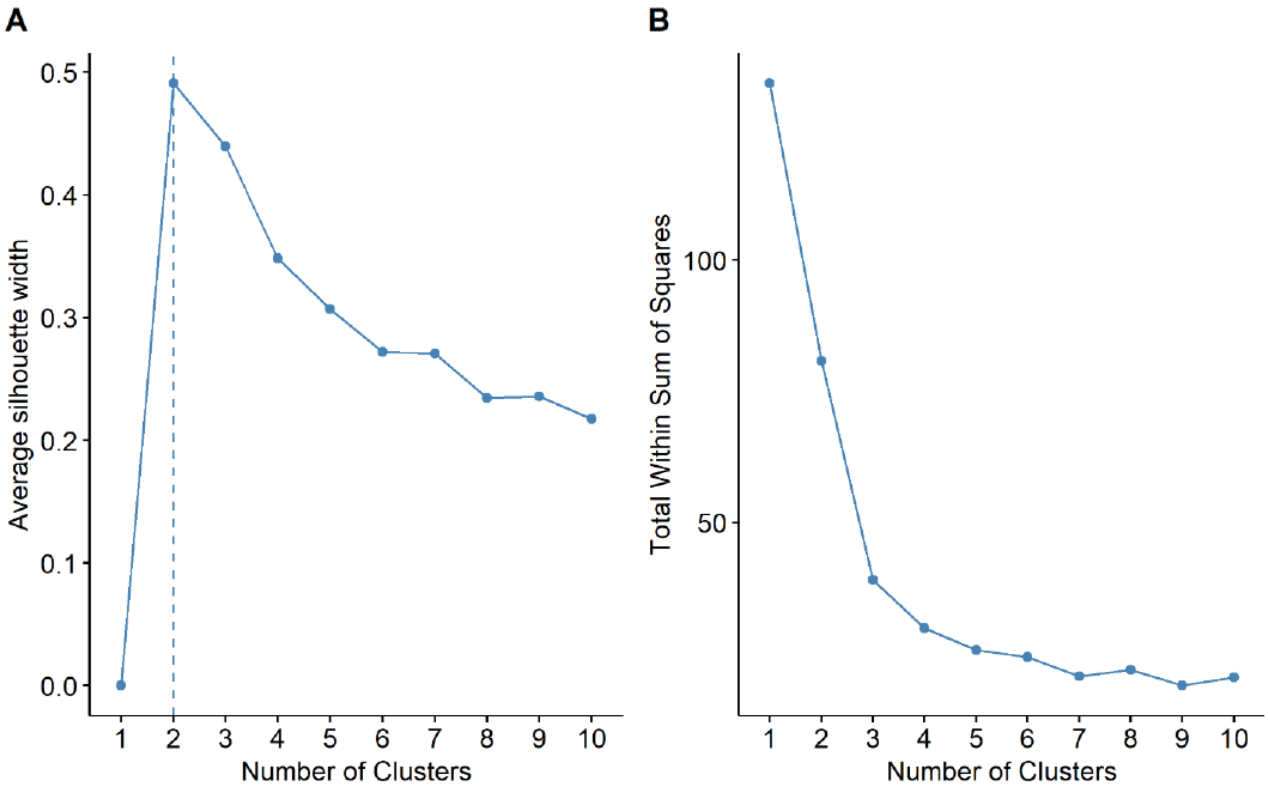

Supplemental Figure 1. Cluster optimization using silhouette scores and within sum of squares. Clusters were estimated using the full TLB profile at baseline (T0) and are not based on TLB metrics. (A) Silhouette provides a measure of how well clusters separate through dimensions, with evaluation based on cluster separation providing two optimally distinct clusters, with only a minimal drop for three clusters. (B) Further dissection of clusters by total within sum of squares (TWSS) shows a significant drop in TWSS when including a 3rd cluster, with monotonic decrease up to k = 7. Based on evaluation of cluster purity and metrics, an optimal cluster size of k = 3 was chosen.

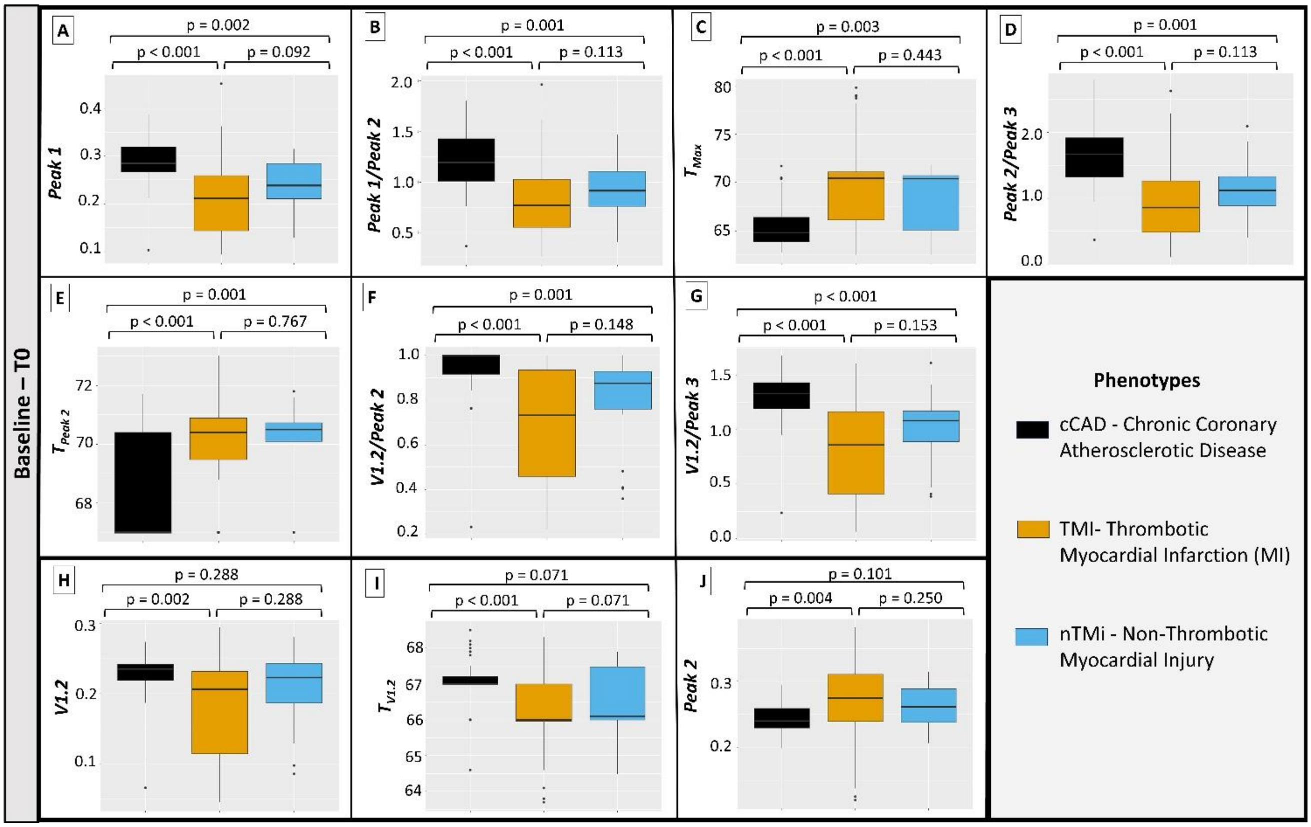

Supplemental Figure 2. TLB profile metrics at baseline (T0) comparing chronic coronary atherosclerotic disease (cCAD), thrombotic myocardial infarction (TMI), and non-thrombotic myocardial injury (nTMi). TLB profile metrics represented in **Panels A** to **G** differentiate acute myocardial injury (TMI and nTMi) from cCAD. **Panels H** to **J** differentiate only TMI from cCAD.

Note: Peak amplitudes corresponding to the temperature region 60-66°C (*Peak 1*) and 67-73°C (*Peak 2*); the temperature of *Peak 2* ($T_{Peak\ 2}$); the ratio of *Peak 1* and *Peak 2* amplitudes (*Peak 1/Peak 2*); the ratio of *Peak 2* and *Peak 3* amplitudes (*Peak 2/Peak 3*); the minimum (valley) between *Peak 1* and *Peak 2* (*V1.2*); temperature of *V1.2* ($T_{V1.2}$); the ratio of *V1.2* and *Peak 2* (*V1.2/Peak 2*); the ratio of *V1.2* and *Peak 3* (*V1.2/Peak 3*); and the temperature of maximum profile amplitude (T_{Max}).

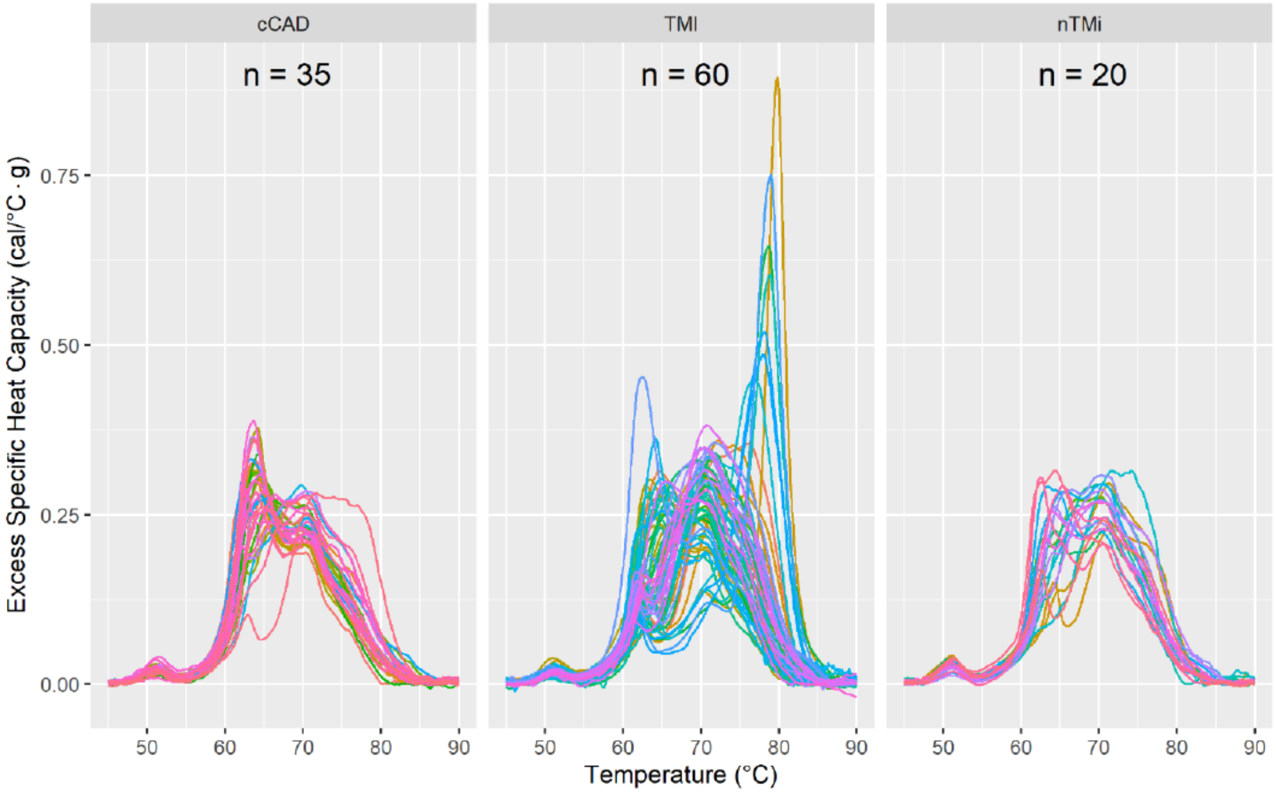

Supplemental Figure 3. TLB profiles at baseline (T0) grouped by clinical phenotype and colored by individual patient. cCAD: chronic coronary atherosclerotic disease, TMI: thrombotic myocardial infarction, nTMi: non-thrombotic myocardial injury.

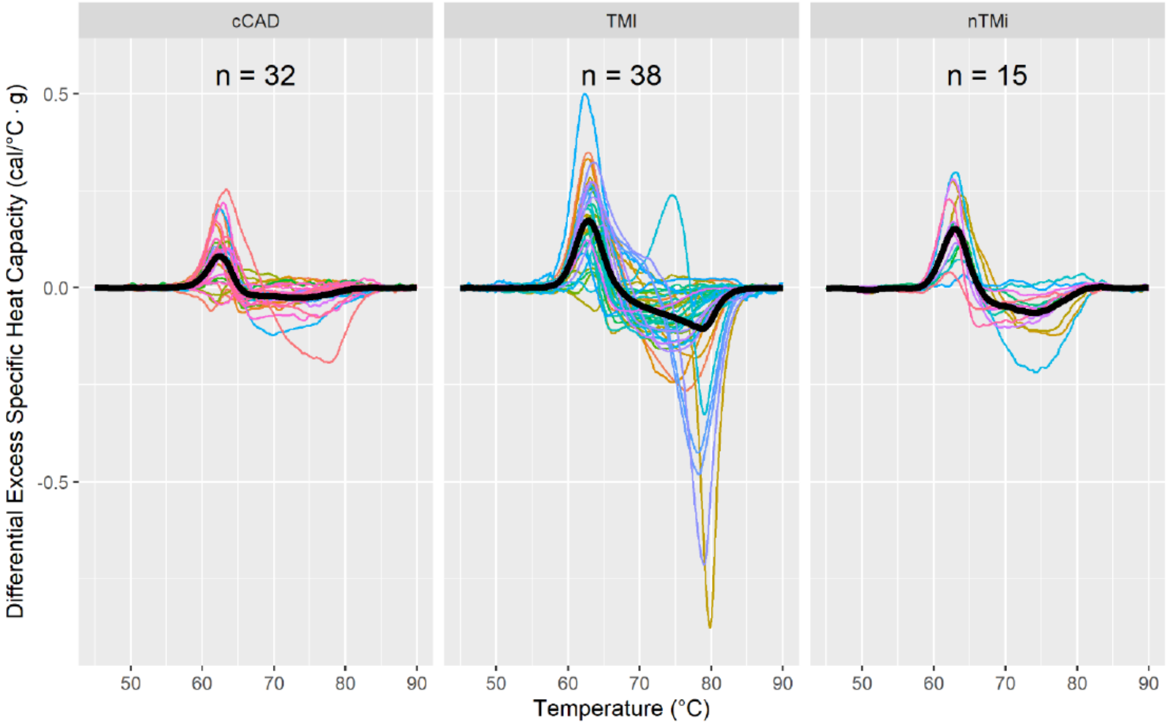

Supplemental Figure 4. Difference TLB profiles between stable cardiac state at 3–12-month follow-up (Tfu) and baseline (T0) colored by individual patient. Mean difference TLB profiles are overlaid (bold black) with sample size listed for all patients having paired TLB profiles (Tfu – T0). cCAD: chronic coronary atherosclerotic disease, TMI: thrombotic myocardial infarction, nTMi: non-thrombotic myocardial injury.
